# Anteroinferior bundle of the acromioclavicular ligament plays a substantial role in the joint function during shoulder elevation and horizontal adduction: a finite element model

**DOI:** 10.1186/s13018-022-02966-0

**Published:** 2022-02-05

**Authors:** Ausberto Velasquez Garcia, Farid Salamé Castillo, Max Ekdahl Giordani, Joaquin Mura Mardones

**Affiliations:** 1 Department of Orthopedic Surgery, Clinica Universidad de los Andes, Av. Plaza 2501, Las Condes, 7620157 Santiago, Chile; 2 Department of Orthopedic Surgery, Hospital Militar de Santiago, Santiago, Chile; 3 Department of Mechanical Engineering, Universidad Tecnica Federico Santa Maria, Santiago, Chile; 4 Department of Orthopedic Surgery, Clinica las Condes, Santiago, Chile

**Keywords:** Acromioclavicular ligament, Finite element analysis, Anteroinferior bundle, Shoulder motion, Acromioclavicular kinematics

## Abstract

**Background:**

Postoperative acromioclavicular (AC) ligament deficiency has been identified as a common cause of failure after isolated coracoclavicular reconstruction. The two-bundle arrangement of the acromioclavicular ligament has recently been reported in histological and anatomical research. In addition, a clear structural advantage of the superoposterior bundle (SPB) over the less consistent anteroinferior bundle (AIB) was also found. However, the current understanding of the function of the acromioclavicular ligament in joint stability is based on uniaxial bone loading experiments and sequential ligament sectioning. Consequently, these rigid biomechanics models do not reproduce the coupled physiological kinematics, neither in the normal joint nor in the postoperative condition. Therefore, our goal was to build a quasi-static finite element model to study the function of the acromioclavicular ligament based on its biomechanical performance patterns using the benefits of computational models.

**Methods:**

A three-dimensional bone model is reconstructed using images from a healthy shoulder. The ligament structures were modeled according to the architecture and dimensions of the bone. The kinematics conditions for the shoulder girdle were determined after the osseous axes aligned to simulate the shoulder elevation in the coronal plane and horizontal adduction. Three patterns evaluated ligament function. The peak von Mises stress values were recorded using a clock model that identified the stress distribution. In addition, the variation in length and displacement of the ligament during shoulder motion were compared using a two-tailed hypotheses test. *P* values < 0.01 were considered statistically significant.

**Results:**

The peak von Mises stress was consistently observed in the AIB at 2:30 in coronal elevation (4.06 MPa) and horizontal adduction (2.32 MPa). Except in the position 2:00, statistically significant higher deformations were identified in the two bundles during shoulder elevation. The highest ligament displacement was observed on the *Y*- and *Z*-axes.

**Conclusions:**

The AIB has the primary role in restricting the acromioclavicular joint during shoulder motion, even though the two bundles of the AC ligament have a complementary mode of action. During horizontal adduction, the SPB appears to prevent anterior and superior translation.

**Supplementary Information:**

The online version contains supplementary material available at 10.1186/s13018-022-02966-0.

## Background

The selection of the ideal reconstruction technique to treat acromioclavicular joint (ACJ) dislocations is still being discussed [1]. In biomechanical studies, anatomical reconstruction of the coracoclavicular (CC) ligaments has demonstrated superior primary stability and load to failure similar to native ligaments compared to non-anatomical reconstructions [2–4]. However, less favorable clinical outcomes have been found in up to 42% of cases with persistent dynamic posterior instability after anatomical CC reconstruction [5]. Postoperative deficiency of the acromioclavicular (AC) ligament has been suggested as one of the potential causes of these failures [6–8].

The role of the acromioclavicular ligament complex (ACLC) in horizontal translation and rotational stability has been published in several studies. Similar to the CC ligaments, the function of the ACLC has been analyzed by selective ligament sectioning and tensile testing [9–11]. Furthermore, in most laboratory-based research, uniaxial external bone loading is commonly applied in horizontal or vertical planes [12] and recently added rotational torque [9–11, 13]. In most experimental models, that rigid bony fixation system simulates an unreal kinematic of the shoulder girdle [14].

Recently, Nolte et al. [15] have shown a consistent quantitative pattern of AC ligament attachment. This morphological description agrees with that reported by Nakazawa et al. [16] in their anatomical and histological study. These authors separated the AC ligament into two distinct bundles—the well-developed superoposterior bundle (SPB) and the anteroinferior bundle (AIB). These morphological descriptions might provide valuable information to support the development of new surgical techniques [8]; however, a better definition of the kinematics of the ACJ and a precise understanding of the individual function of the AC ligament during shoulder motion is needed to improve anatomic ligament reconstructions [14].

The technology used in finite element analysis (FEA) plays a valuable role in joint mechanics research in the orthopedic field [17]. FEA has been increasingly used to analyze ligament function, yield information, and dynamic variations in stress distribution that are impossible to replicate under other laboratory conditions [14, 17, 18].

To our knowledge, there have been no studies that consider stress distribution as a predictor of the function of the AC ligament in shoulder motion. Therefore, the primary purpose of this study was to evaluate the role of the AC ligament bundles by assessing the stress distribution, deformation, and ligament displacement during coronal plane shoulder elevation and horizontal adduction. In shoulder motion, the clavicle translates more posteriorly when the humerus is elevated in the coronal plane than in the sagittal or scapular planes [19]. Furthermore, abnormal posterior translation of the clavicle has been associated with AC ligament insufficiency and poorer postoperative clinical results [5]. In addition, we reconstruct the humeral position of the cross-body adduction stress test by recreating the horizontal adduction motion [20]. This physical examination test is very sensitive in identifying pathological states of ACJ on clinical [21] and radiographic examination [22]. In addition, the authors hypothesized that the SPB of the AC ligament would provide the greatest stability compared to AIB.

## Methods

### Building of a three-dimensional bone model

The study was conducted according to the Helsinki declaration and its modifications. We obtained ethical approval from our institutional Medical Ethics Committee. Digital Imaging and Communications in Medicine were obtained from a 0.67 mm wide computed tomography scan of a healthy male volunteer (25 years old; height 175 cm; weight 78 kg). Data were segmented with 3D Slicer 4.11 (Boston, USA) using automatic threshold-limited and manual identification to obtain the boundaries of each bone of the shoulder girdle, thoracic spine, and sternum. After bone delineation, the surface geometries were exported into Meshmixer (Autodesk Inc, San Rafael, CA, USA) in STL format. Then, the ribs and other unwanted structures were removed, and the irregularities of the surface model were repaired and smoothed. The solid model of the bones was initially meshed into high-quality tetrahedral elements and then imported into ANSYS 2020R2 (Pennsylvania, USA) to perform the subsequent steps and finite element analysis.

### Modeling of ligament structures

**Fig. 1:**
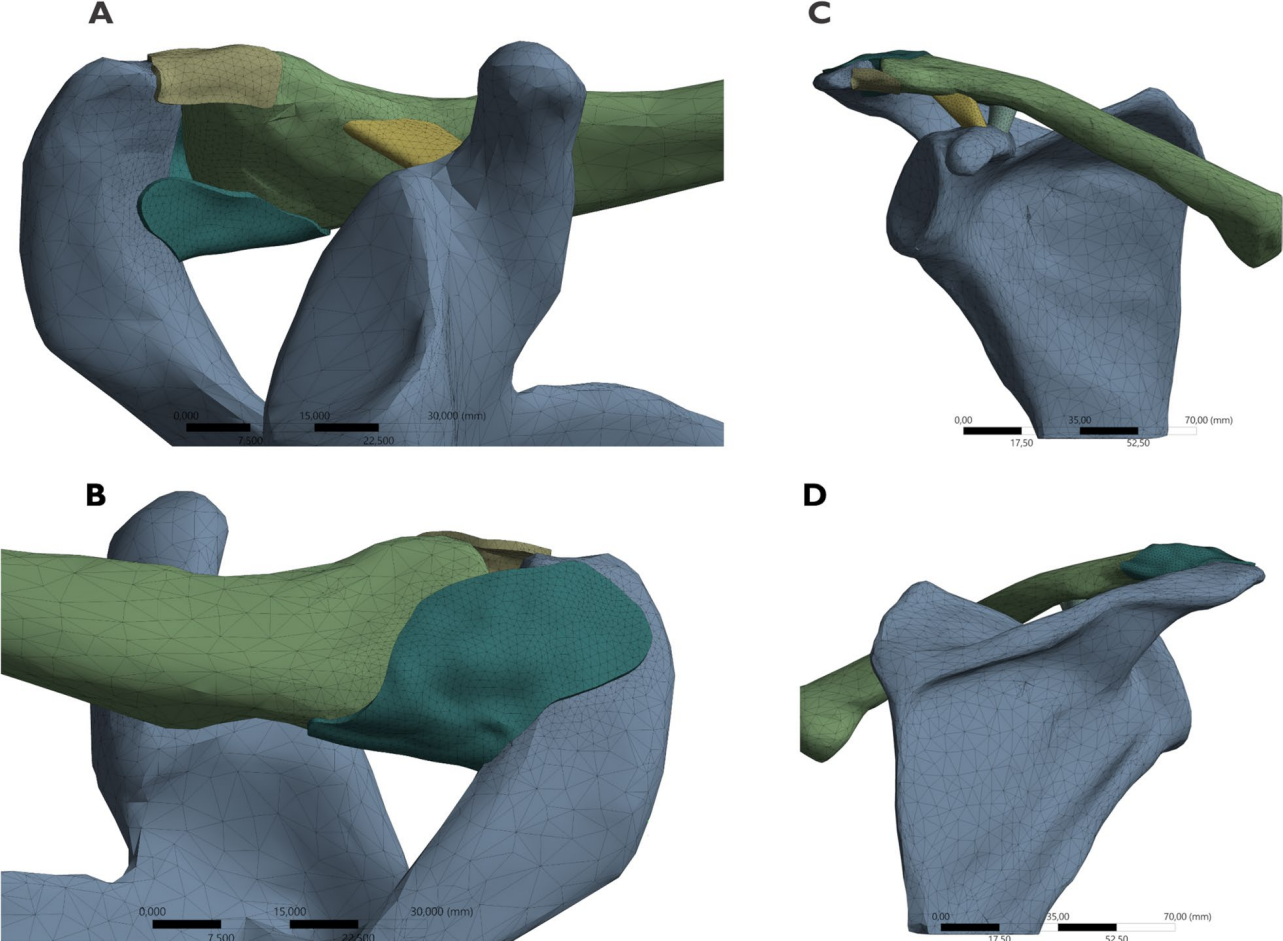
Finite element model of the clavicle, scapula, and acromioclavicular ligament. The model includes the coracoclavicular ligaments. **A** Inferior view. **B** Superoposterior view. **C** Anterior view. **D** Posterior view

The ligaments were developed virtually from the insertion footprint's delimitation and the edges' connection using software tools. The SPB and the AIB of the AC ligament were reconstructed based on recent anatomical descriptions [15, 16]. Type 2 was selected for our model among AIB variants because it was the most prevalent subtype. This type of bundle extends from the anterior to the inferior surface of the AC joint capsule. However, it does not cover the inferior surface of the joint entirely [16]. Therefore, the insertion sites of the AC ligament bundles were carefully determined. First, the distance from the cartilage edge of each bone to the nearest edge of the footprint was measured. Second, the farthest insertion of each bundle was delimited according to the mean values of the footprint width corresponding to the superior, posterior, inferior, and anterior aspects of each bone [15]. After measuring and delimiting the footprints, their areas were calculated. The bundles were then created and converted into solid models (Fig. 1). In addition, the CC ligaments were also reconstructed to simulate more physiological models. We used the dimensions and location of the insertion sites according to previous studies [23–25].

### Meshing and material properties

For the ligaments, a convergence test was conducted to refine the mesh. Thus, linear tetrahedral elements with a maximum size of 0.5 mm allow element-wise stress evaluations. Bone structures were assumed to be homogeneous, isotropic, and rigid materials [26, 27]. The mechanical behavior of the ligaments was assigned as homogeneous, isotropic, and hyperelastic coefficients. Accordingly, the Arruda–Boyce hyperelastic model [28] was selected to simulate the high incompressible deformations in the ligament tissue. The values applied in this model were obtained from previous experimental measurements [29]. The material properties used in our model are summarized in Table 1.

**Table 1:** Definition of the material properties of tissues in the FEM

	Young's Modulus (MPa)	Poisson ratio	Density (kg/m^3^)	References
	Hyperelastic constants—Saint Venant–Kirchhoff			
Clavicle	11 000	0.3	1 800	Iwamoto et al. [26]
Scapula	11 000	0.3	1 800	Metan et al. [27]
	Hyperelastic constants—Arruda–Boyce model			
AC and CC ligaments	µ [MPa]	0.982		Correia. [29]
	λ_*m*_	6.999		
	*D*_1_ (MPa^−1^)	0.211		

*FEM* finite element model, *AC* acromioclavicular, *CC* coracoclavicular

To validate these parameters, we used a dumb-bell test piece subjected to the standards of the German Institute for standardization normative (DIN 53504-S3A:1994) [see Additional file 1]. The constitutive model was assessed using a custom Python script [see Additional file 2] for the uniaxial tensile test on the dumb-bell piece for our model and compared against the exact solution for the properties of the mechanical parameters assigned to the ligaments (Table 1). Unfortunately, due to the novelty of the design, there is no analogous model available; nonetheless, we conducted an indirect validation method by comparing our results with previous experiments. Subsequently, AC joint kinematics under external moments were predicted using our FE model while preserving the features of our constitutive model and compared with those obtained in native joints from cadaver experiments reported by Morikawa et al. [10] and Beitzel et al. [30] [see Additional File 3].

### Axis alignment

**Fig. 2:**
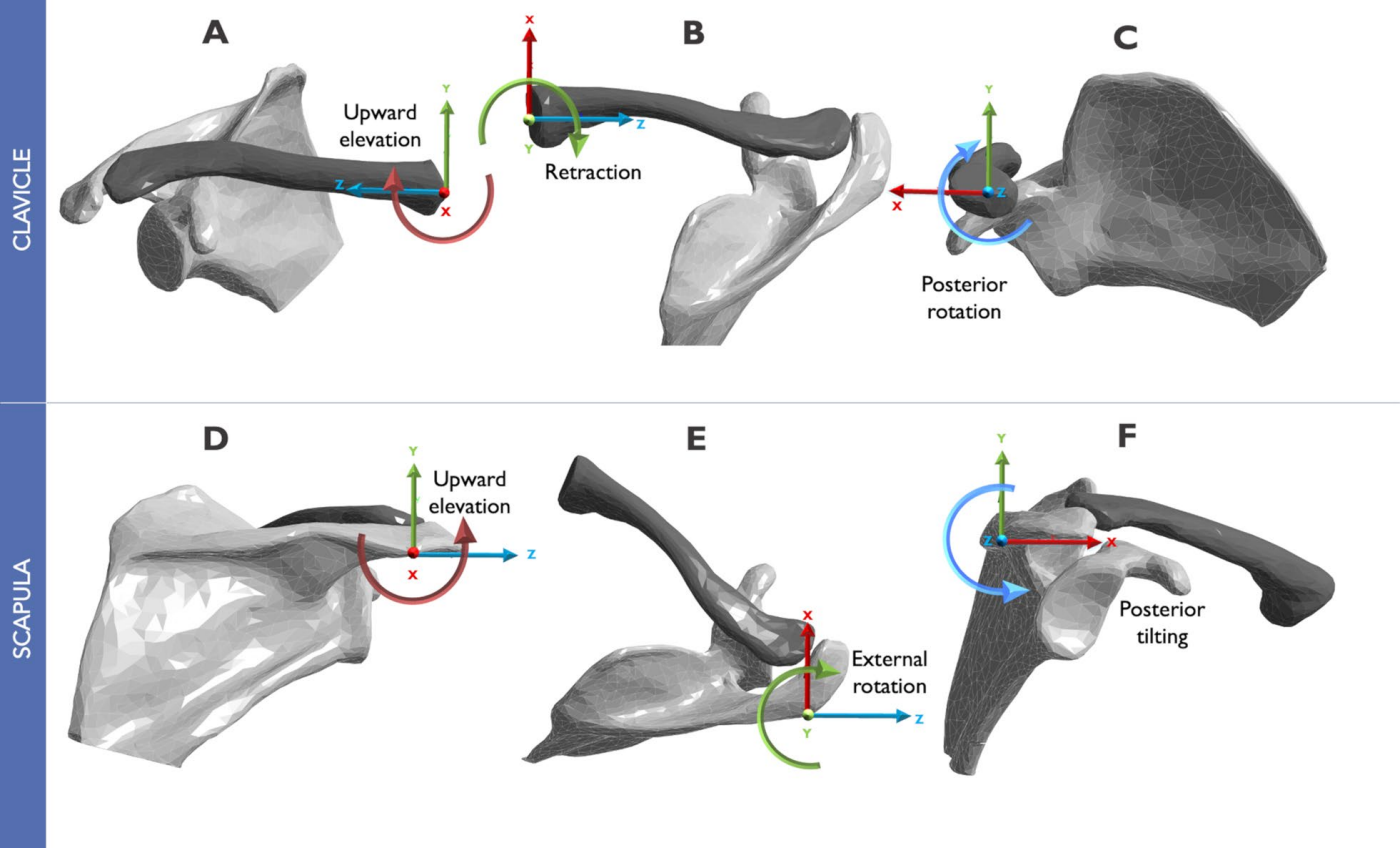
Local coordinate system of clavicle (**A**–**C**) and scapula (**E**, **F**). Definition of sternoclavicular (**A**–**C**) and scapulothoracic (**D**–**F**) motion. Using a 3D coordinate system and Cardan or Euler angles, following International Society of Biomechanics recommendations [31]

The landmarks on the shoulder girdle and spine were initially identified. The 3D local coordinate system for the thorax, clavicle, and scapula was established at the reference points following the recommendations of the International Society of Biomechanics for the upper extremity [31]. The most ventral point on the sternoclavicular (SC) joint and the most laterodorsal point of the acromion were defined as the origin of the clavicle and scapula coordinate system, respectively. Therefore, the SC and scapulothoracic (ST) movements were standardized at these points (Fig. 2).

Angular rotations of the clavicle and scapula in the reference position were assessed using Cardan or Euler angles. Gravity significantly affects the angular rotations and positions of the shoulder girdle. The standing position results in more clavicle retraction and less elevation than the supine position, while the upward scapular rotation, anterior tilt, and internal rotation are lower than in the supine position [32]. Since the bone model was built from a CT scan in the supine position, these gravitational effects were incorporated before running the simulation. The values of our bone model's angular rotations and orientations outside the standard deviation reported by Matsumura et al. [32] were modified by translation and rotation of the bony structures to replicate the orientation of the shoulder girdle in a standing position. Consequently, these changes made the bone orientation compatible with the initial kinematics values used [20].

### Shoulder girdle kinematics

Two different computational models were created. The first model was used to simulate shoulder elevation in the coronal plane, and the second model was used to replicate horizontal shoulder adduction. Consequently, shoulder motion was computer-generated in different planes according to the humerothoracic position. Therefore, the shoulder coronal elevation plane was defined as 0° of humerothoracic elevation, whereas the adduction plane was defined as 90° of humerothoracic elevation (axial plane). The simulation is designed to keep the palm downward throughout the horizontal adduction, similar to the cross-body adduction test. Quasi-static and nonlinear FEA was performed in both models.

**Fig. 3:**
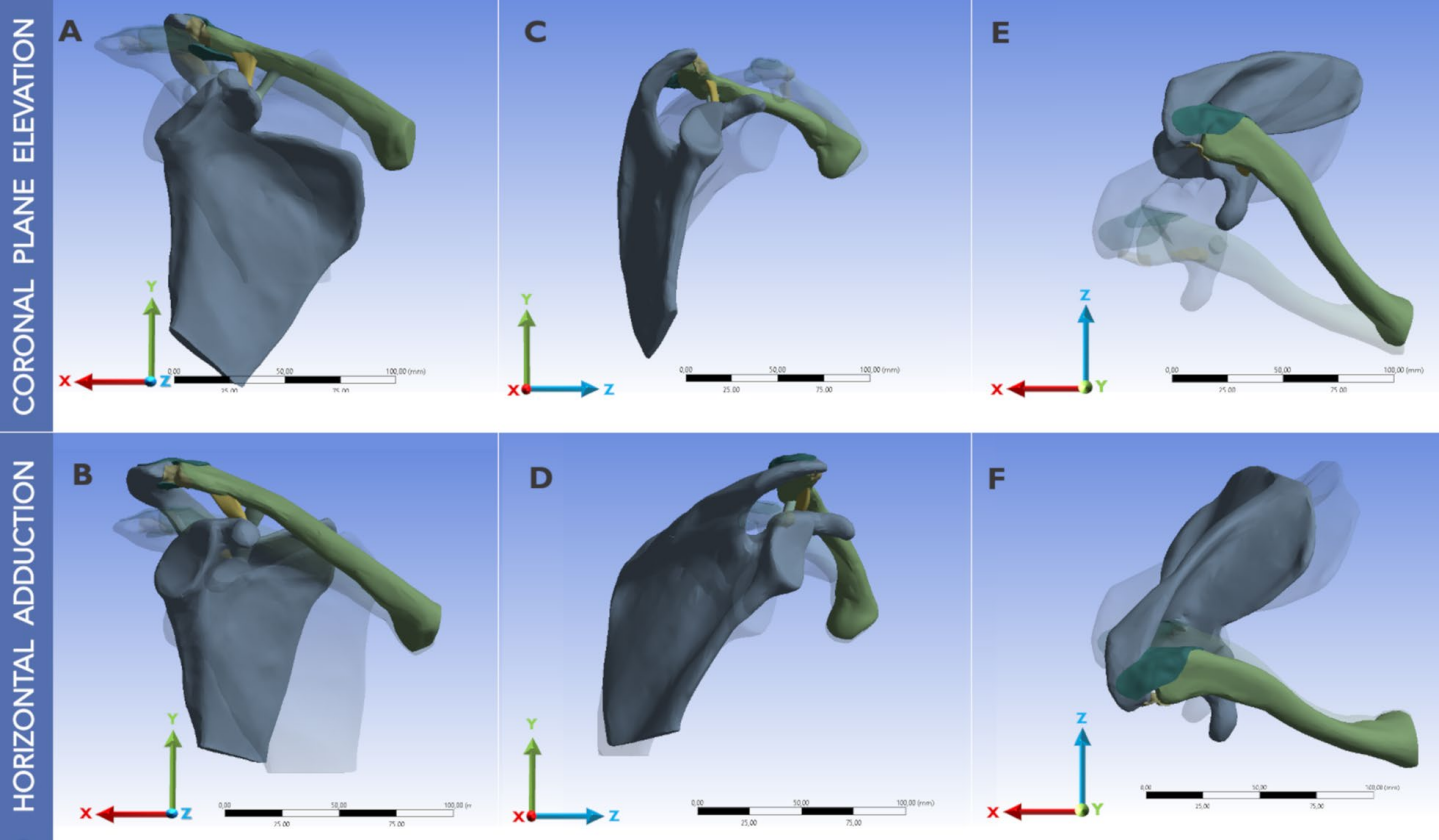
Simulation of sternoclavicular and scapulothoracic kinematics during coronal plane elevation and horizontal adduction in a right shoulder. **A**, **B** Anterior view. **C**, **D** Lateral view. **E**, **F** Superior view. The phantom images represent the initial three-dimensional orientation of the bones, while the dark images illustrate the final position at the end of the movement. A global coordinate system is established; thus, the ligament deformation and displacement can be measured and expressed

SC and ST motions were then replicated in the models. For this purpose, 3D angular rotations were progressively assigned from the origin of the coordinate system for both the scapula and the clavicle according to the corresponding angle of coronal elevation or horizontal adduction for the first and second models, respectively. Kinematic changes in scapular and clavicular motions during humerus motion have been reported in previous studies [20]. Therefore, we replicated normal SC and ST kinematics (Fig. 3) during shoulder elevation angles from 20° to 120° (coronal plane) and adduction angles from 20° to 100° (axial plane). These values have been described as normal ranges of motion in daily life [33].

### Contact and boundary conditions

The frictionless type of contact was assigned for the AC and SC joints. In addition, bones and ligaments were assembled using a type of bonded contact for connections between the two structures, so that the reciprocal motion in the nodes in the *X*-, *Y*-, and *Z*- axes were forbidden. Five degrees of freedom (DOF) were allowed for the scapular bone. Three DOF for rotational motion around the *X*-, *Y*-, and *Z*- axes allowing: upward/downward rotation, internal/external rotation and, anterior/posterior tilt, respectively (Fig. 2). The two DOF for the translational motion were elevation/depression, protraction/retraction. The surface of the sternal joint of the clavicle was fixed.

Consequently, in our simulations, three DOF were permitted for clavicle rotational motion around the *X*-, *Y*-, and *Z*- axes allowing: upward/downward elevation, retraction/protraction, and posterior/anterior rotation, respectively (Fig. 2). Furthermore, the motions occurring in the SC joint and the AC joint are meant to describe the movement of the clavicle relative to the thorax and the scapula relative to the clavicle, respectively. Finally, the motion that occurs in the ST joint describes the motion of the scapula relative to the thorax.

### Outcome measures

**Fig. 4:**
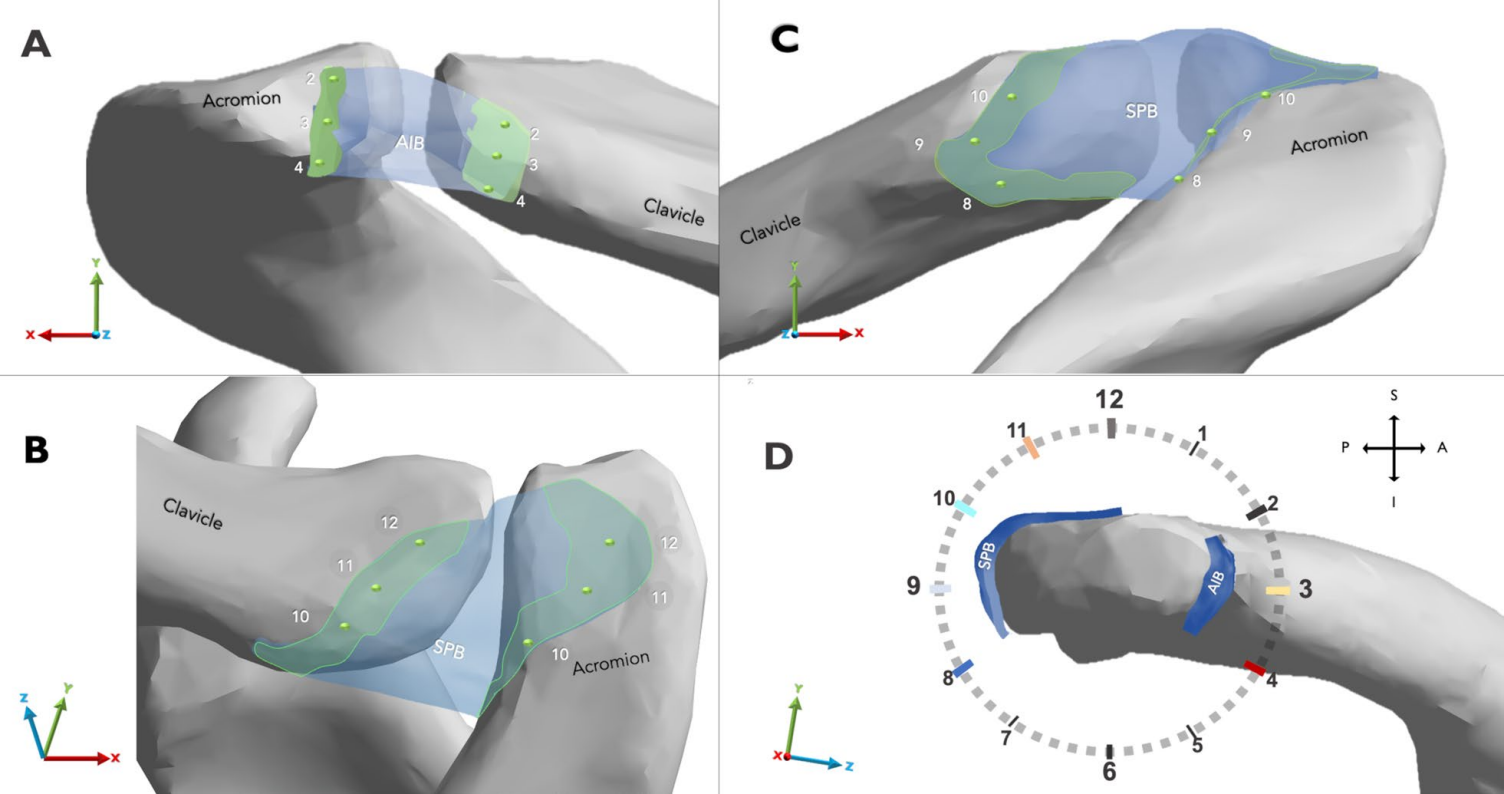
Schematic representation of the footprint area of the acromioclavicular ligament in a right shoulder. The registration nodes are arranged and paired according to the clock model [15]. The green area represents the bone attachment of the AC ligament. **A** Anterior view of the ACJ. **B** Superoposterior view of the ACJ. **C** Posterior view of the ACJ. **D** Parasagittal view of the clavicle showing the PSB, AIB, and the clock model. *ACJ* acromioclavicular joint, *AC* acromioclavicular, *SPB* superoposterior bundle, *AIB* anteroinferior bundle

The ligaments were studied using three measurements; peak von Mises stress, displacement, and deformation. Peak von Mises stress was expressed in megapascals (MPa), representing the distribution of the total energy within a specific ligament considering its biomechanical characteristics. Hence, it can be correlated to the failure load and predict yielding [34]. Stress was monitored within each virtual ligament in a time-dependent result every 0.1 s (equivalent to 1 degree of humeral motion). Simulated arm movements were recorded under standard earth gravity (*G* = 9.806 m/s^2^). A right shoulder's clockface model [15] was used to precisely the AC ligament's stress location (Fig. 4).

The deformation in the ligaments was reported in millimeters (mean ± SD) and determined from morphological changes in length. Sixteen nodes were selected on the superficial aspect of the AC ligament and paired according to the clockface model. They were located in the center of the corresponding ligament attachment at the acromion and clavicle site. Therefore, each pair of nodes was located at the clock positions of 2:00, 3:00, and 4:00 to evaluate the AIB. For calculations on the SPB, the selected nodes were paired every hour from 8:00 to 12:00 (Fig. 4).

Furthermore, we used millimeters to describe the magnitude of displacement of the bundles on the three axes (*X*-, *Y*-, *Z*-) of the global coordinate system throughout the simulations (Fig. 4). The magnitudes of ligament length deformation and three-axis ligament displacement were calculated using the Pythagorean theorem; therefore, the Euclidian distances between the paired nodes were calculated for each shoulder motion position to measure deformation. Subsequently, the ligament length variation ratio (Δ Distance) was expressed as percentages obtained by dividing the difference between the final and initial Euclidian distances by the initial distance.

### Statistical analysis

All statistical analyses were conducted using SPSS 16.0 software (22.0, IBM, Armonk, NY, USA). The peak von Mises stress of the ligaments was recorded using absolute values at seven different positions between 20 and 120° of coronal plane shoulder elevation. Similarly, six different positions between 20 and 100° were used for assessing horizontal shoulder adduction.

Furthermore, the mean values and their standard deviation were calculated for the variation of ligament length and ligament displacement on each of the three axes (*X*-, *Y*-, *Z*-). Two-tailed hypotheses were used to compare the differences between ligament length variation and ligament displacement on the three axes in the two shoulder movements. *P* values < 0.05 were regarded statistically significant.

## Results

During the verification test, the theoretically predicted values of the dumb-bell strain–stress curve [35] showed high correspondence with the data obtained in our experimental model. In general, the findings obtained after indirect validation demonstrated the high accuracy of the current FE model compared to previously well-developed experimental biomechanics tests [see Additional Files 3 and 4]. The number of elements, nodes, material properties of our FEM, and ligament footprint areas of the AC and CC ligaments are summarized in Table 2.

**Table 2:** Structural description of the tissues in the FEM. Number of nodes and elements, footprint areas of the ligaments

				Footprint area (mm^3^)		
		# Elements	# Nodes	Coracoid	Clavicle	Acromion
Bones	Clavicle	28,053	17,184	–	–	–
	Scapula	27,231	46,204	–	–	–
AC Ligament	AIB	1604	3189	–	28.9	18.2
	SPB	7736	15,107	–	125.9	154.8
CC Ligaments	Trapezoid	1774	3183	30.0	56.3	–
	Conoid	1360	2417	37.6	46	–

*FEM* finite element model, *AC* acromioclavicular, *CC* coracoclavicular, *AIB* anteroinferior bundle, SPB superoposterior bundle

### Peak von Mises stress

**Fig. 5:**
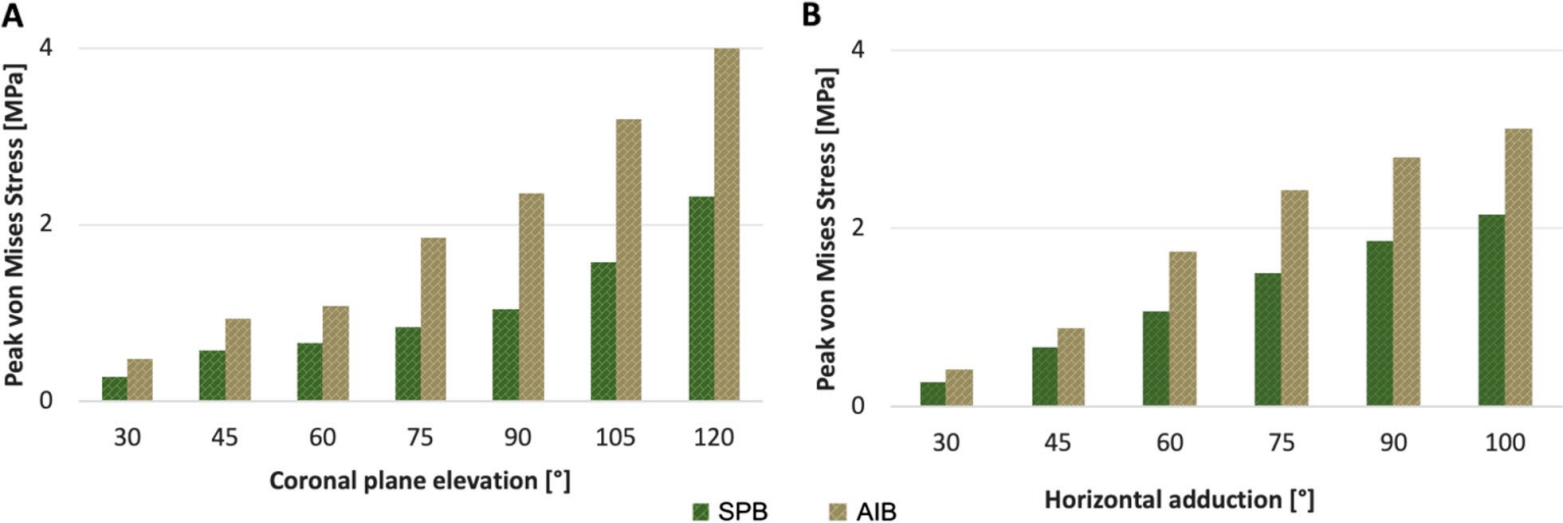
Peak von Mises stress [MPa] distribution of the AC ligament during shoulder motion. **A** Coronal plane elevation. **B** Horizontal adduction. *SPB* superoposterior bundle. *AIB* anteroinferior bundle

**Fig. 6:**
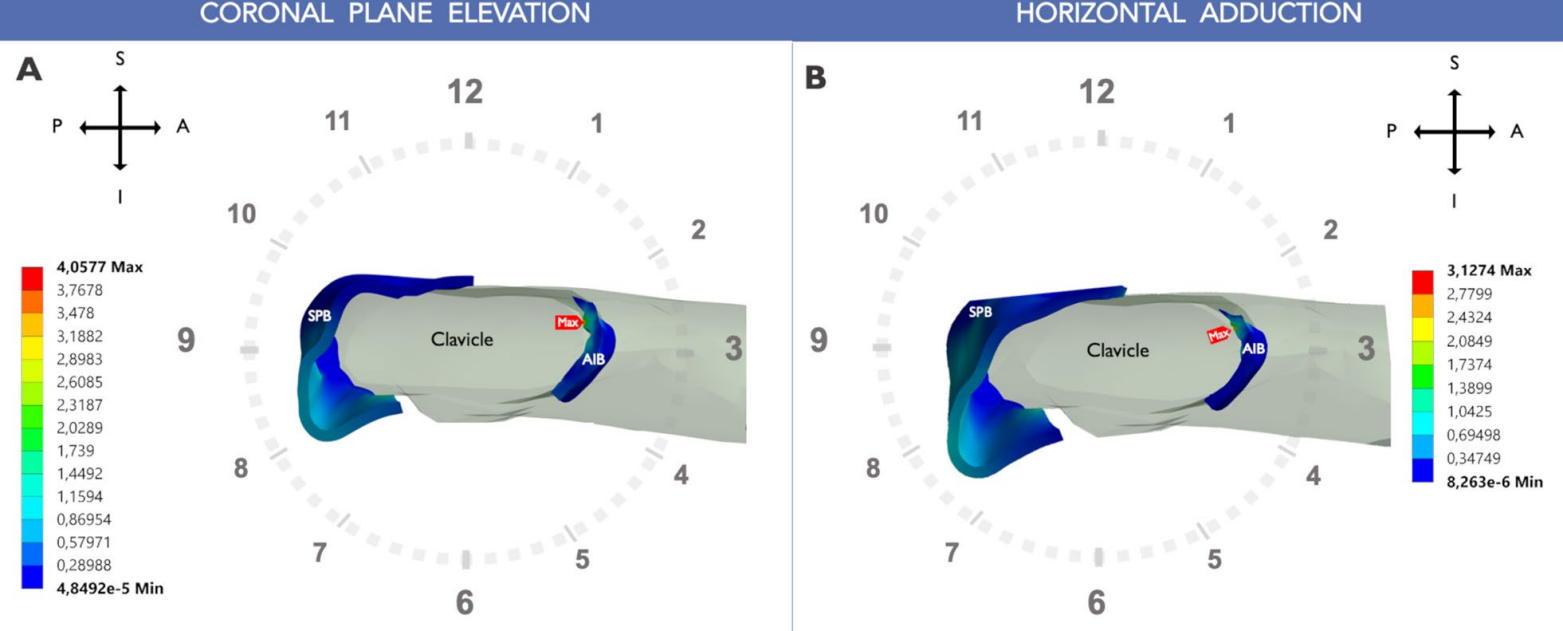
Peak von Mises stress (MPa) distribution in the AC ligament at the end of the shoulder motion. Parasagittal view a right clavicle showing the highest stress level in the AC ligament. **A** Coronal plane elevation. **B** Horizontal adduction. *SPB* superoposterior bundle, *AIB* anteroinferior bundle. *MAX* maximum stress. *A* anterior, *P* posterior, *S* superior, *I* inferior

**Fig. 7:**
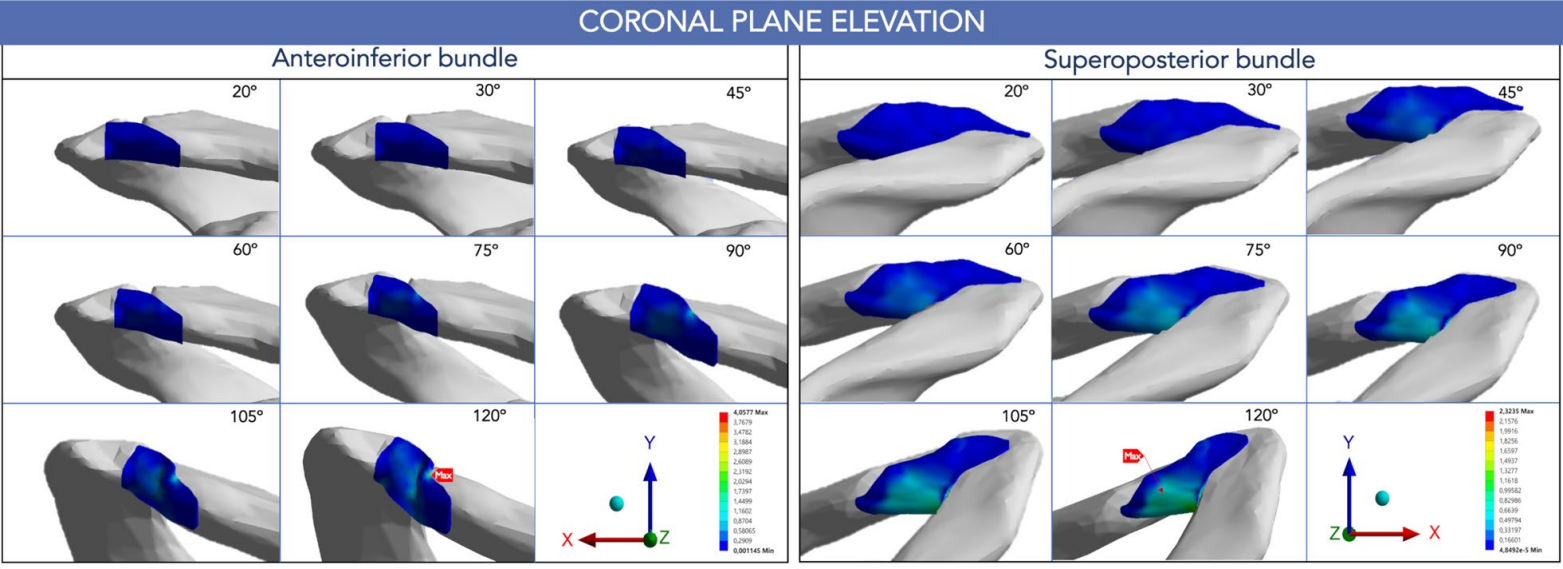
Peak von Mises stress of the acromioclavicular ligament in shoulder coronal plane elevation

During coronal plane shoulder elevation, the peak von Mises stress in the AC ligament bundles had a similar pattern of linear increase as the degree of humerus elevation progressed. However, the stresses were not uniformly distributed between the two bundles. The highest value (4.06 MPa) was observed at the AIB clavicular insertion site (at 2:30, using the right shoulder clockface model) at 120° of shoulder elevation (Figs. 5, 6). The SPB carried 43% less stress at 120° elevation (2.32 MPa) than the AIB (Fig. 5). The peak SPB stress was located at the posterior aspect of the clavicular insertion site (at 9:00). The stress distribution in the AC ligament along the coronal elevation is shown in Fig. 7.

**Fig. 8:**
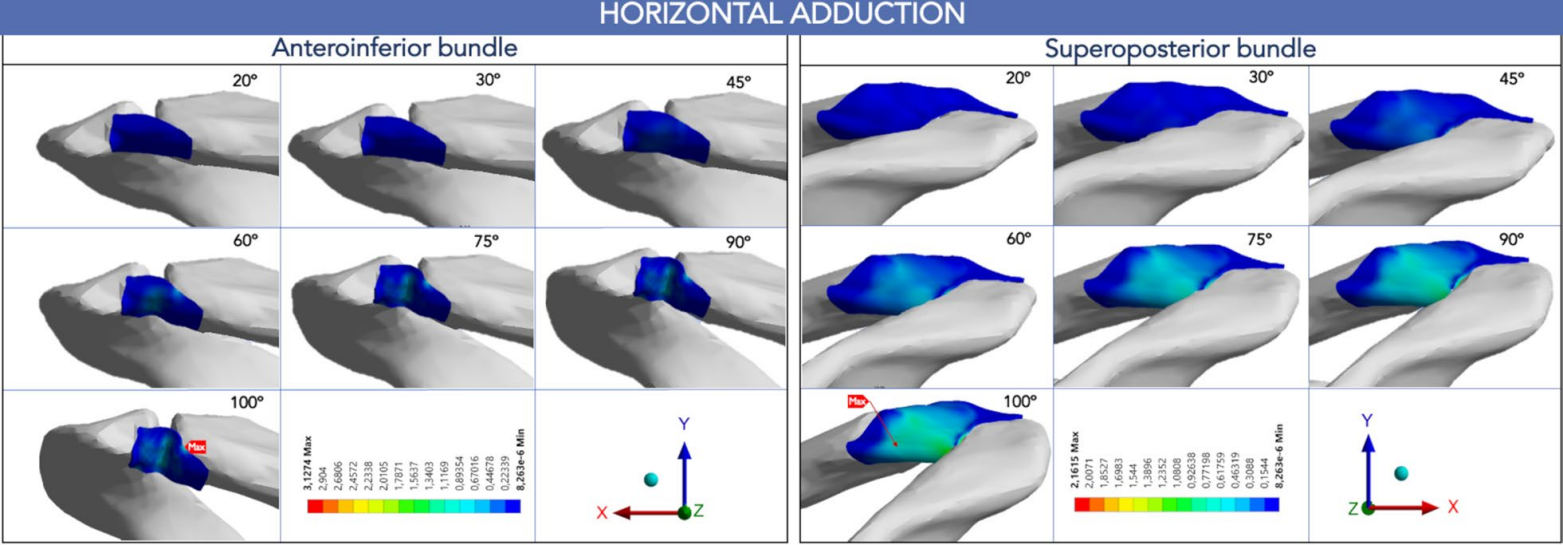
Peak von Mises stress of the acromioclavicular ligament in shoulder horizontal adduction

In horizontal shoulder adduction, the highest stress value was 3.13 MPa observed at the end of the motion located in the AIB at 2:30. This peak of stress was 44% higher than the maximum value of the SPB (2.16 MPa at 10:00). Similar to shoulder elevation, the maximal von Mises stress was located at the clavicular insertion of the AIB during the six horizontal adduction positions that were recorded (Figs. 5, 8).

### AIB deformation

**Fig. 9:**
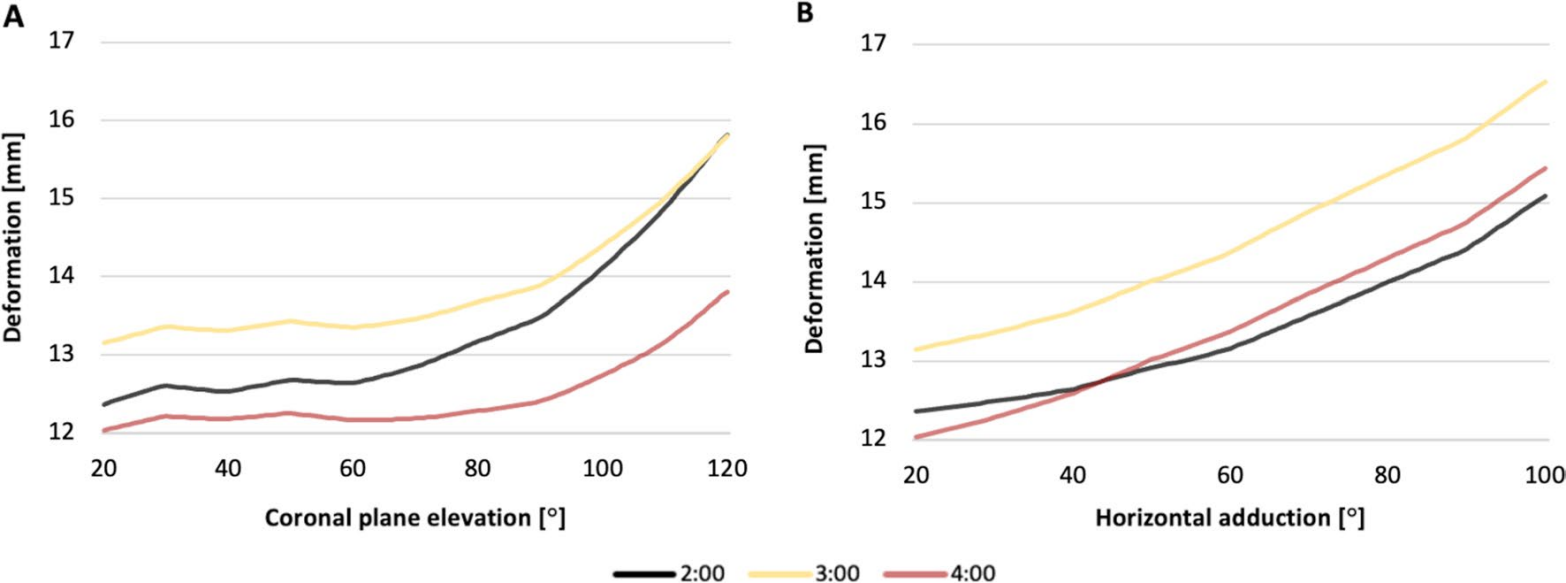
Deformation of the anteroinferior bundle throughout shoulder motion. **A** Coronal plane elevation. **B** Horizontal adduction. The deformation was obtained by recording the length change between the paired reference nodes using the Pythagorean theorem

The virtual length of the bundles is defined by the distance between the center of its insertional areas. For the AIB, the initial virtual length was 12.37 mm, 13.15 mm, and 12.04 mm at the 2:00, 3:00, and 4:00 clock positions, respectively. The AIB increased its length linearly throughout the horizontal adduction. In contrast, the ligament length remained relatively constant until 80° of coronal shoulder elevation (Fig. 9). However, AIB increased its length by 22%, 26%, and 28% at the end of the horizontal adduction of the shoulder, compared to its initial length at the clock positions 2:00, 3:00, and 4:00, respectively (Table 3).

**Table 3:** AC ligament length (mm) during shoulder motion

	Coronal plane elevation		Horizontal adduction		
Location	AIB length (mm)	Δ Distance %	AIB length (mm)	Δ Distance %	*P* value
2:00	13.31 ± 0.94 (12.37–15.82)	+ 27	13.21 ± 1.66 (12.37–15.08)	+ 22	0.656
3:00	13.84 ± 0.69 (13.15–15.79)	+ 20	14.36 ± 1.86 (13.15–16.54)	+ 26	< 0.001
4:00	12.46 ± 0.43 (12.04–13.81)	+ 15	13.33 ± 1.76 (12.04–15.43)	+ 28	< 0.001
	SPB length (mm)		SPB length (mm)		
8:00	14.35 ± 0.80 (15.79–12.88)	− 23	14.96 ± 0.48 (15.79–14.14)	− 12	< 0.001
9:00	14.70 ± 0.92 (16.28–12.94)	− 26	15.18 ± 0.61 (16.28–14.19)	− 15	< 0.001
10:00	16.68 ± 1.45 (15.55–20.30)	+ 31	14.65 ± 0.64 (15.55–13.62)	− 14	< 0.001
11:00	15.94 ± 0.88 (15.46–18.22)	+ 18	14.53 ± 0.40 (15.46–14.16)	− 9	< 0.001
12:00	17.51 ± 0.94 (16.42–20.33)	+ 24	16.47 ± 0.23 (16.42–17.09)	+ 4	< 0.001

Data are expressed as mean ± standard deviation (initial length – final length). The initial length was set as a reference with 100%. Δ Distance% was calculated by dividing the final and initial lengths by the initial length. The *P* values were calculated to compare the elevation of the coronal plane (from 20° to 120°) and horizontal adduction (from 20° to 100°)

*AC* acromioclavicular ligament, *AIB* anteroinferior bundle, *SPB* superoposterior bundle

Furthermore, statistically significant differences were found between the mean length of the AIB during the shoulder elevation compared to the mean length during horizontal adduction, at 3:00 (13.84 ± 0.69 mm vs. 14.36 ± 1.86 mm) and 4:00 position (12.46 ± 0.43 mm vs. 13.33 ± 1.76 mm) *P* = 0.00, respectively. Meanwhile, there were no statistical differences in mean length at 2:00 position between shoulder elevation and horizontal adduction (13.31 ± 0.94 mm vs. 13.21 ± 1.66) *P* = 0.656 (Table 3).

### SPB deformation

For the SPB, the initial length was 15.79 mm, 16.28 mm, 15.55 mm, 15.46 mm, and 17.51 mm at 8:00, 9:00, 10:00, 11:00, and 12:00 positions, respectively (Table 3). Unlike the AIB, the SPB did not show a consistent lengthening according to its reference nodes. Compared to the initial measure, the distance between the center of the footprint at positions 8:00 and 9:00 decreased by 23% and 26% during shoulder elevation, respectively. A similar shortening was observed in the SPB (from 8:00 to 11:00 positions) at the end of the horizontal adduction motion. In contrast, the SPB length at 12:00 increased 24% and 4% at the end of the shoulder elevation and horizontal adduction, respectively (Fig. 9). However, the most significant lengthening of the SPB was observed at the 10:00 clock position after shoulder elevation.

The mean length along the 5 different clock positions studied in the SPB differed significantly between the two shoulder kinematic models. The highest mean value (16.68 ± 1.45 mm) was observed at 10:00 during shoulder elevation. The overall data are shown in Table 3.

### AIB displacement

**Fig. 10:**
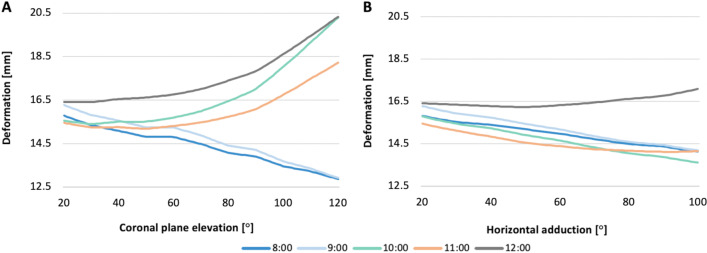
Deformation of the superoposterior bundle throughout shoulder motion. **A** Coronal plane elevation. **B** Horizontal adduction. The deformation was obtained by recording the length change between the paired reference nodes using the Pythagorean theorem

Component displacements have directional (positive and negatives) values as they can be described in relation to an axis of the global coordinate system. The mean displacement along the three axes is shown in Fig. 10. The displacement of the *Y*-component of the AIB during shoulder elevation and horizontal adduction was a mean of 4.823 ± 0.762 mm and 3.937 ± 0.063 mm (*P* = 0.004), respectively. This suggested that the mean displacement of AIB was cephalic during both simulations.

Although the displacement of the *Z*-component during coronal elevation was higher (− 4.367 ± 0.762 mm) than that during adduction (− 1.933 ± 0.094 mm), no significant differences were found *P* = 0.041. As a result, the dorsal displacement of the AIB was similar during both shoulder motions. Likewise, no statistical differences were found in the mean displacement along the X-component in both simulations (3.100 ± 0.572 mm vs. 3.180 ± 0.166) *P* = 0.087.

### SPB displacement

The overall displacement of the SPB was primarily along the *Y*- and *Z*-components. The displacement on the *Z*-axis was significantly different (*P* = 0.006) between the two models. Regarding the direction, the mean displacement of the SPB along the *Z*-component during coronal elevation was dorsal during elevation (− 1.312 ± 0.440 mm) and volar during adduction (3.220 ± 1.784 mm). Additionally, between the two models of shoulder motions, the AIB *X*- and *Y*-axis displacements were not significantly different (*P* = 0.767, *P* = 0.171, respectively). The general data are shown in Fig. 10.

## Discussion

The most important finding of this study is that the bundles of the AC ligament have an unequal load-sharing behavior in both the elevation of the shoulder in the coronal plane and the horizontal adduction. During our evaluation, we consistently found a pattern of higher von Mises stress values in the clavicular footprint of the AIB, between 2:00 to 2:30. On the contrary, the SPB showed a lower stress, and its maximum values were consistently located on the clavicular side, between 9:00 and 10:00, according to the clockface model of the right shoulder described previously [15]. Maier et al. [8] reported the pattern of AC ligament injury in acute ACJ lesions. They found that more than 70% of the injuries were associated with detachment of the ligament in the clavicular area. The outcome in the present study suggests that the AIB plays a significant role compared to the SPB by controlling the clavicular strut function in the ACJ kinematics, according to the levels of energy that it bears in the motions tested.

Our simulations were based on the normal AC ligament's anatomy and the articular kinematics' descriptions. Recently, Nakazawa et al. [16] have detailed the morphology of the AC ligament. SPB is a well-defined capsular thickening consistently found in all specimens with a 30° oblique orientation. The attachments of this bundle originate from the superior, posterior, and inferior aspects of the clavicle. The insertion on the superior acromion in all specimens marks the oblique orientation of this structure.

In contrast, AIB was a thinner structure classified into three types according to the variations of its footprint sites and dimensions [16]. Based on the prevalence reported, we reconstructed type 2. In addition, Nolte et al. [15] determined the footprint width of the AC joint capsule and ligaments. The widest insertional footprint (6.6 mm) was measured in the posterosuperior quadrant of the clavicular (limits between 8:00 and 12:00 in a clockface model) and the acromial sides, corresponding to the PSB.

Morphological descriptions suggest that the SPB plays a crucial role in the ACJ function. Several published studies have determined the function of different areas of the AC ligament [9–11, 36–38]. Kurata et al. demonstrated that the SPB, in conjunction with the CC ligaments, plays an important role in supporting the superior translation of the ACJ compared to the AIB. After sequential sectioning of the AC ligament and uniaxial loading tests, the superior displacement increased > 50% after SPB sectioning [37].

In contrast, Dyrna et al. [38] demonstrated that the anterior segment of the ACJ capsule provides the highest stability. Conversely, they evaluated the biomechanical response under rotational loading and posterior translation rather than vertical displacement. In these experiments, the amplitude of the joint motion increased significantly after the dissection of this structure. Furthermore, 91% of native posterior translation stability was restored after ACJ anterior bracing reconstruction in a cadaver model that evaluated horizontal stability [11].

Similarly, Morikawa et al. [10] evaluated the specific regional contributions (anterior, superior, and posterior segments) of the superior half of ACLC. They evaluated posterior translation and rotational stability after sequential sectioning of the ACLC. The authors found a significant increase in resistance to posterior translation after suturing the anterior third of the AC ligament (*P* = 0.025). Furthermore, the resistance torque increased significantly only after suturing the anterior and posterior regions, unlike any other combination of regions (*P* < 0.001). These results are comparable to the highest stress distribution that we observed.

However, several biomechanical experiments have not restored the joint condition before injury [9, 30, 38]. According to these studies, a closer approximation of normal kinematics was obtained only after reconstructing the entire ACLC and not by reconstructing other specific regions of the AC ligament [9, 30, 37, 38]. Nevertheless, those results do not rigorously apply to the actual postoperative state since they did not reproduce the physiological ACJ motion. Consequently, comparing these outcomes with our data should be done with caution due to the different experimental conditions.

In the present study, we identified the individual function of the AC ligament during shoulder motion, and we located areas that yielded significantly more stability. These findings might be used as a guide to improve the location of fixation points in reconstruction techniques. Due to the complex 3D motion in the shoulder girdle, ligament function may not be adequately assessed by uniaxial translational or rotational loads in a fixed model [14], as is the case in almost all previous studies [9, 30, 38].

On the contrary, we aimed to simulate the 3D motion of the normal shoulder girdle to evaluate the kinematics and mechanical behaviors of the ligaments under physiological conditions. During shoulder motion, ST motion is generated by a mechanical coupling at the SC joint and ACJ rather than isolated uniaxial rotations or angular displacements [19, 39, 40], which rarely occurs in real-life [41]. Accordingly, the coupling theory is crucial to developing biomechanical models to explain functional and movement patterns [40]. Thus, to better evaluate the function of the AC ligament and create a more realistic model, we aimed to simulate the ST, the SC, and ACJ kinematics [14, 20, 41].

Several studies have investigated the 3D shoulder girdle kinematics by various methods [19, 39, 40, 42]. Oki et al. [20] evaluated shoulder girdle kinematics using electromagnetic tracking devices in cadaver models. The scapula rotated internally and then externally, tilted posteriorly, and rotated upward (6°, 10°, 37°, respectively); meanwhile, the clavicle rotated posteriorly and upward, and retracted posteriorly (17°, 16°, 18° respectively) when the humerus is elevated in the coronal plane. On the contrary, they showed that the scapula rotates internally compared to the clavicle in horizontal adduction, and the upward rotation is significantly lower than in elevation [20]. Those coupling angular rotations are challenging to be replicated in conventional experiments [14, 20, 41].

According to our results, the virtual AC ligament is exposed to an unequal strain during shoulder elevation and horizontal adduction due to the pattern of deformations demonstrated. During coronal plane elevation, the AIB showed its highest enlengthen (27%) at the 2:00 position. Furthermore, the most significant increase in AIB was observed from 90° of shoulder elevation and was also related to its higher stress concentration, especially at 2:00. Following an in-vivo ACJ kinematic analysis, Sahara et al. [43] described a change in clavicular translation in the horizontal plane when the shoulder exceeds 90° of abduction. Their research found that from 0 to 90° of shoulder abduction, the clavicle translates posteriorly, while from 90° of shoulder abduction, the clavicle moves anteriorly. The authors suggested that the dominant muscular traction force of the superior trapezius causes a posterior clavicular displacement between 0 and 90° of shoulder abduction. In contrast, the traction force of the anterior deltoid is higher from 90° of shoulder elevation, moving the clavicle anteriorly. Consequently, our results suggest a primary role for the AIB in constraining the posterior but particularly anterior displacement of the clavicle during shoulder abduction.

At the 3:00 and 4:00 clock positions, the virtual AIB was exposed to significantly higher strain during horizontal adduction than at shoulder elevation. However, the higher level of stress at the 2:00 position that the AIB bears throughout horizontal adduction suggests its ability to prevent AC dissociation compared to the other positions of the bundle. It is important to note that the AIB did not demonstrate nearly isometric distance in any position studied.

During coronal plane elevation, the reference nodes on the SPB moved away at 10:00, 11:00, and 12:00 positions (31, 18, 24% respectively), especially after 90° of coronal elevation. In contrast, the nodes of the SPB at 8:00 and 9:00 were approached from 60° of shoulder elevation. Therefore, as long as the highest stress distribution in the AIB during this motion occurred at the 9:00 position, we hypothesized that the approach between the footprints does not necessarily reflect slack in the ligament. On the contrary, the AC rotational motion during shoulder elevation could create a torsional force on the SPB that creates significant stress to the fibers but does not increase the distance between the center of the footprint. This assumption is supported because the ACJ rotates significantly during shoulder abduction. Previous studies reported between 15 to 35° of normal rotation of the ACJ [43–45]. The current study found 20° ACJ relative rotation during coronal plane shoulder elevation [see Additional file 5].

**Fig. 11:**
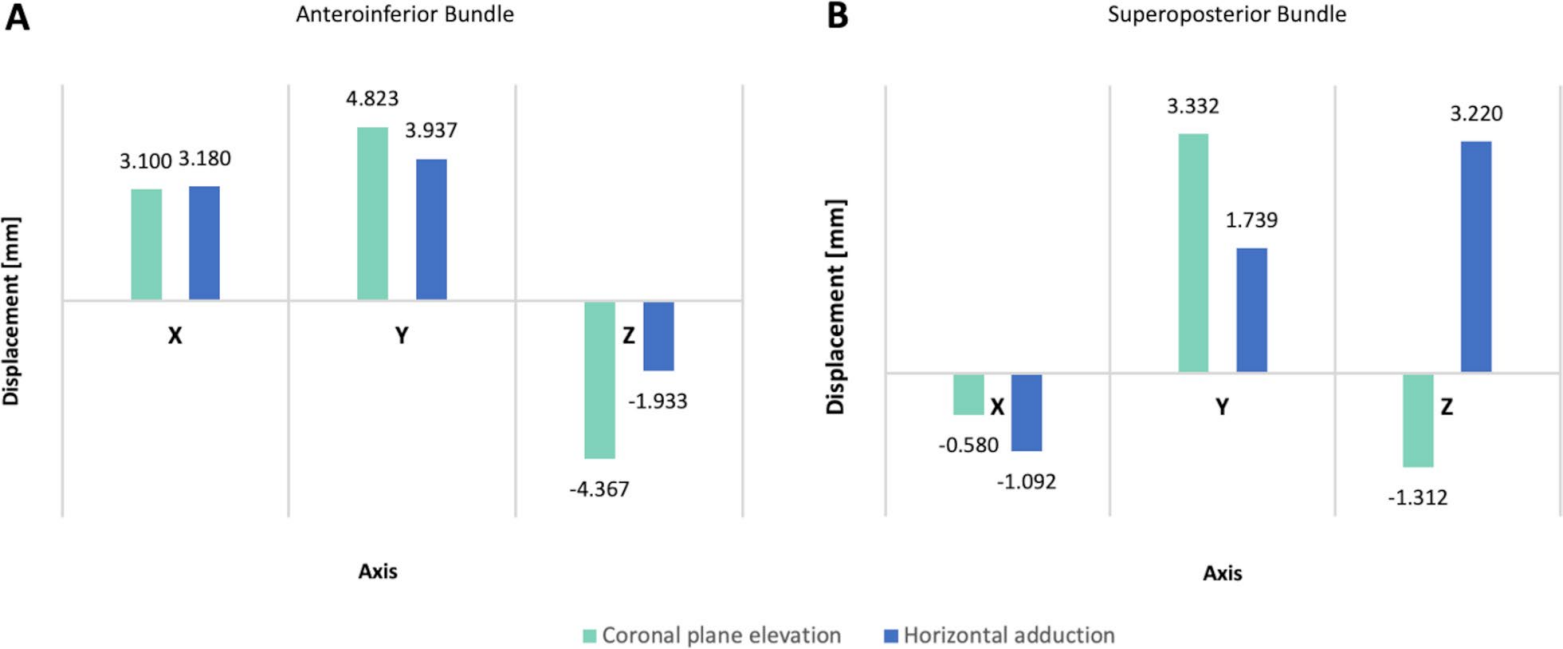
Acromioclavicular ligament bundle's displacement (mm) on the three axes, according to the global coordinate system. **A** Anteroinferior bundle. **B** Superoposterior bundle

In addition, the displacement of the AIB was mainly on the *Y*-and *Z*-axes during coronal elevation. Thus, according to the direction of the reference nodes, the AIB controls the articular stability against the posterosuperior translation of the ACJ. On the contrary, during horizontal adduction, the overall displacement of the AIB was primarily in the superior and lateral directions (Fig. 10). Likewise, the SPB demonstrated a similar path of displacement on the *Y*-and *Z*-axes along shoulder elevation (Fig. 11). However, a lower magnitude in displacement and a lower peak von Mises stress distribution suggest a secondary stabilizer role in the physiologic kinematics of this motion.

In contrast, the primarily SPB displacement occurred on the *Z*-axis during horizontal adduction. An anterior direction of the displacement of the SPB indicates that this bundle may constraint the ACJ against anterior loadings. Furthermore, the SPB seems to play a complementary role in restricting the superior translation. To our knowledge, there is not a single study that assesses the stress distribution, deformation, and displacement of the AC ligament in a quasi-static or even rigid model. In this context, little is known about the stress patterns of the AC ligament to establish straight comparisons.

This study has several limitations. First, FEA has intrinsic restrictions, such as simplified boundary conditions and material properties, which can affect the numerical simulation results. Although our model allowed five DOF for the scapula, three DOF for the clavicle, and only restrained the translation of the sternal surface of the clavicle, boundary conditions constructed on muscle loadings might be even more realistic. However, we believe that the results would not differ significantly because SC rotation was fully allowed in our models; thus, the function of the clavicular strut between the scapula and the sternum is preserved. ﻿In addition, we assumed that the ligaments were hyperelastic and incompressible materials, although they are viscoelastic and compressible.

For this reason, rather than using only absolute values as a reference, we compared stress distribution patterns. Therefore, we also reconstructed the CC ligaments to incorporate their stabilizing effect into the model. Consequently, the numerical stress values measured in the AC ligament were closer to reality. Nonetheless, there is no indication that incompressibility influences experimental outcomes [46, 47].

Second, we assumed that the footprints would not be 100 percent accurate compared to a patient-specific model. Consequently, they were reproduced as precisely as possible, using data from anatomical descriptions and a reference of marginal bone ridges. Third, the simulated shoulder movements did not fully replicate the theoretical range of motion. Thus, we cannot accurately predict ligament behavior beyond 120° of shoulder elevation and 100° of horizontal adduction. However, the ligament stress pattern did not follow a trend that appeared to modify the body of the conclusions if we were able to extend the range of motion.

Fourth, we only reconstructed a type 2 AIB. In other smaller-size ligament variations, the magnitude of the stress distribution could be affected; nevertheless, in those circumstances, the component of the ACLC located in the anteroinferior aspect of the ACJ could bear the stress loading correspondingly, as occurred in our model. In addition, many authors have reported that the anterior region of the ACLC has a significant role in joint stability, although their experimental settings were different [9, 38]. Therefore, it is unlikely to obtain different results if the anterior capsule is preserved.

Fifth, only one healthy adult shoulder girdle joint was built; this may not fully represent the complex situation under several pathological conditions and may be insufficient to standardize the results provided. Furthermore, investigations of the biomechanics of different anatomical morphologies are worthy of study from a scientific point of view. However, since this study focuses on the function of the AC ligament based on the kinematic characteristics of the shoulder girdle and the consistent anatomical relationship between bones and ligaments previously reported [15, 16, 24, 25], we used a single representative bone model for this analysis. Although it does not illustrate bone structural variations, it can demonstrate ligament biomechanics patterns within the limitations mentioned above. Finally, we have not considered the joint constraint effect of fascia in our model, and its impact on stability is still uncertain. As a result, more research is needed to investigate these topics, including biomechanical and clinical studies.

Contrary to our hypothesis, the AIB has shown a primary role in maintaining the stability of the ACJ during shoulder coronal plane elevation and horizontal adduction. The peak von Mises stress was greater in the AIB throughout the shoulder motion. According to the clock model, the maximum stresses were supported in the 2:00 and 3:00 locations of the bundle. A secondary role was consistently observed in the SPB, notably at the 9:00 and 10:00 positions.

## Conclusions

Although the two bundles of the AC ligament function in a complementary mode to maintain the kinematics of the ACJ coupling, the AIB plays the primary role in joint constraint throughout the shoulder motion examined. Furthermore, the SPB appears to help avoid excessive anterior and superior translation, particularly during horizontal adduction.

## Supplementary Information

**Additional file 1**: Dumbbell plane generated by Autodesk Inventor.tif. Test geometry (DIN 53504-S3A:1994). The supports are added at the ends to simulate an adhesion condition to a structural steel surface (*E* = 200 [*Gpa*] and* v* = 0.3[-]). The model was embedded at its top, and a fixed displacement was applied at its bottom, according to the boundary requirements established.

**Additional file 2**: Python Script.tif. Script for calculating the theoretical stress value for hyperelastic Arruda–Boyce materials [28].

**Additional file 3**: Model validation.pdf. The model was indirectly validated by comparing the kinematic behavior with previously published cadaver biomechanical studies.

**Additional file 4**: Stress vs. strain simulation and theoretical calculation.tif. The stress and strain values in the axial direction obtained from the FEM were compared with the theoretical data [35]. The discrepancies between the two results are negligible up to a strain of 1.33 mm, indicating that the model's behavior in terms of stresses is as predicted.

**Additional file 5**: Acromioclavicular joint relative rotation during shoulder motion.tif. The acromioclavicular joint relative rotation reached 20° at 120° of shoulder elevation in the coronal plane.

## Data Availability

The data underlying the current study will be shared by the corresponding author on reasonable request.

## Abbreviations

AC: Acromioclavicular
ACJ: Acromioclavicular joint
ACLC: Acromioclavicular ligament complex
AIB: Anteroinferior bundle
CC: Coracoclavicular
DIN: German Institute for Standardization
DOF: Degree of freedom
FEA: Finite element analysis
MPa: Megapascals
SC: Sternoclavicular
SD: Standard deviation
SPB: Superoposterior bundle
ST: Scapulothoracic
