# Supplementary figures and images for: Anteroinferior bundle of the acromioclavicular ligament plays a substantial role in the joint function during shoulder elevation and horizontal adduction: a finite element model
Source: J Orthop Surg Res. 2022 Feb 5;17:73. doi: 10.1186/s13018-022-02966-0 (PMC8818233; doi:10.1186/s13018-022-02966-0)

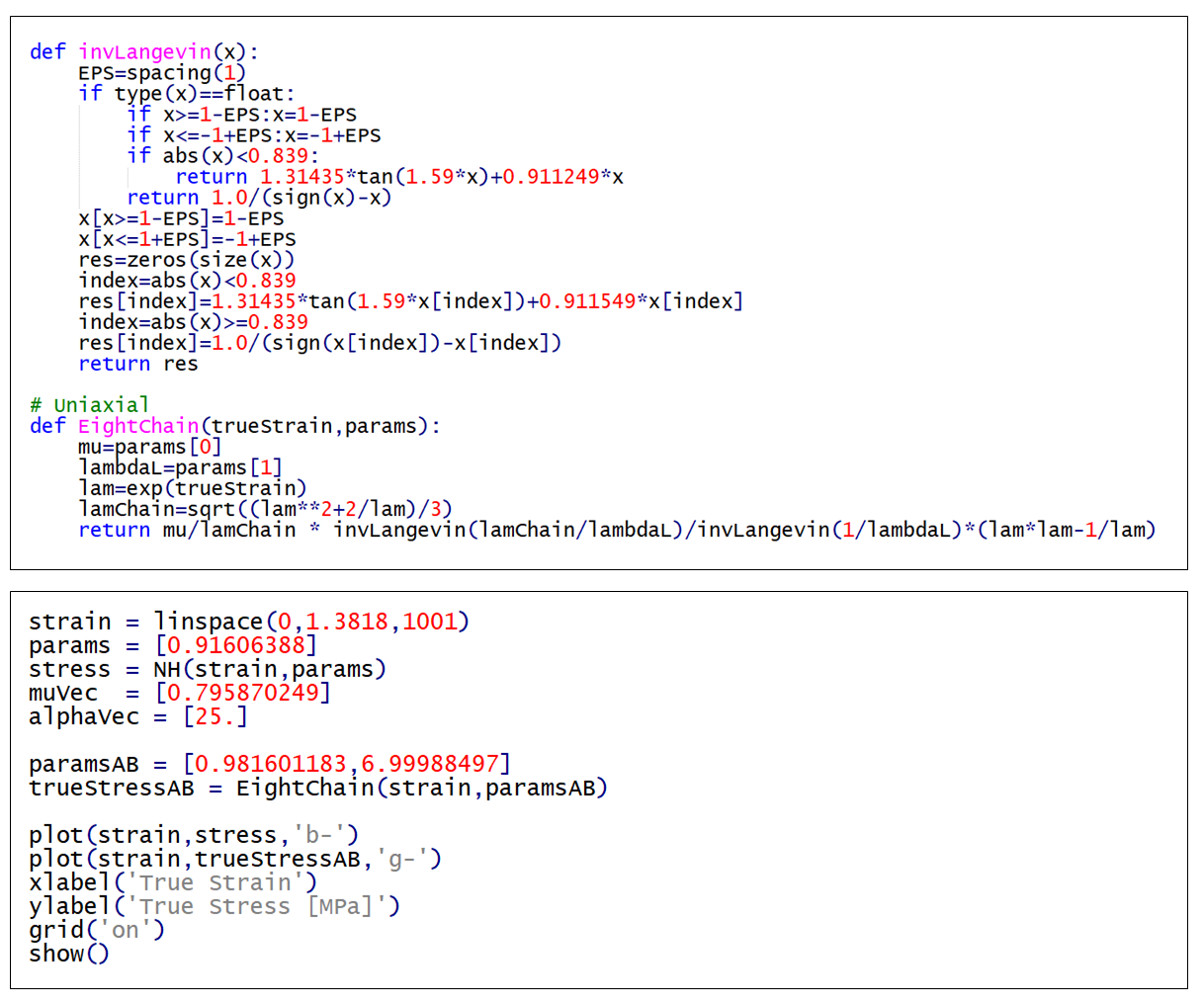

Supplement: Supplementary file 2 — Additional file 2: Python Script.tif. Script for calculating the theoretical stress value for hyperelastic Arruda–Boyce materials [28]. [file 13018_2022_2966_MOESM2_ESM.tif]

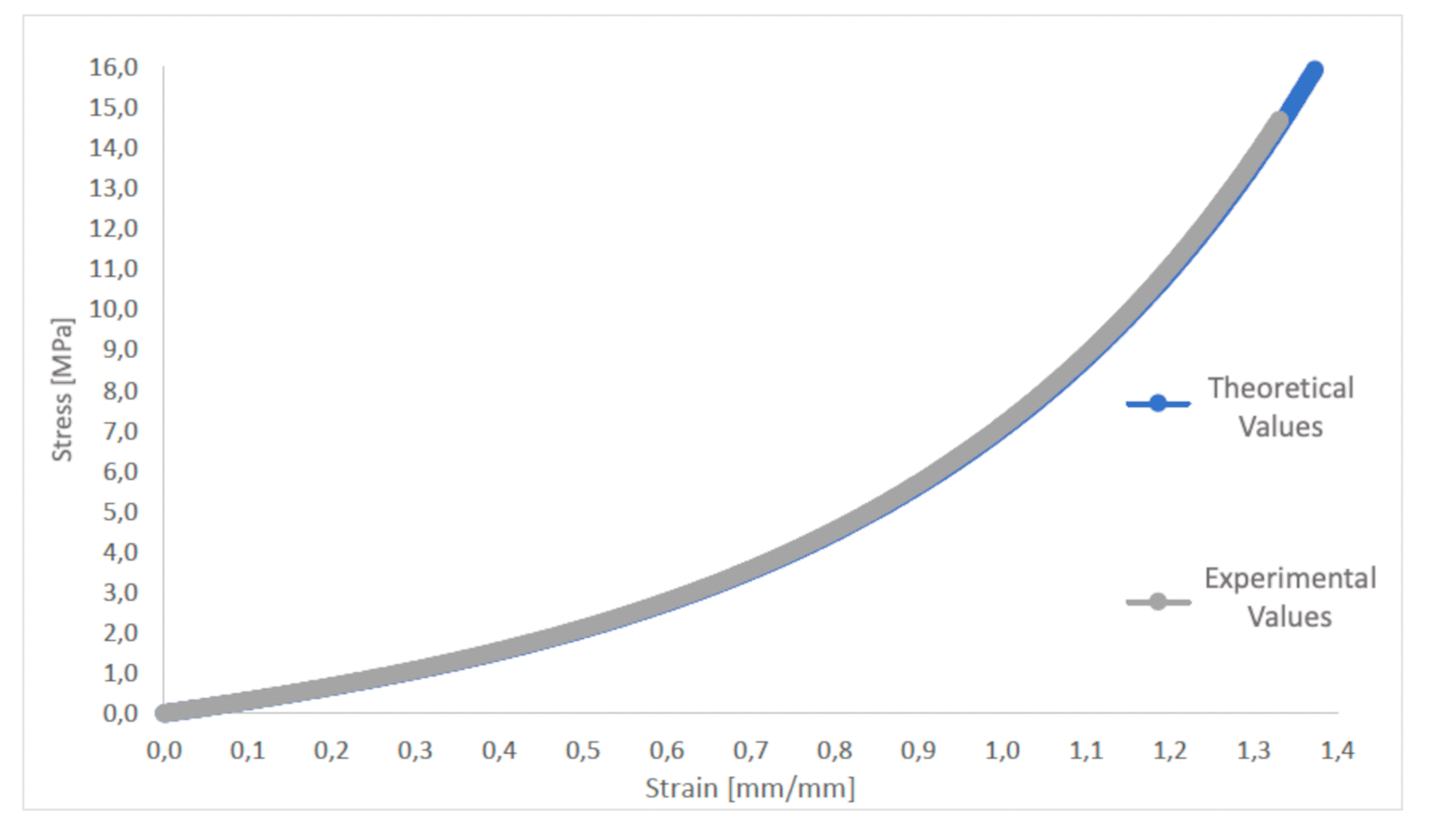

Supplement: Supplementary file 4 — Additional file 4: Stress vs. strain simulation and theoretical calculation.tif. The stress and strain values in the axial direction obtained from the FEM were compared with the theoretical data [35]. The discrepancies between the two results are negligible up to a strain of 1.33 mm, indicating that the model's behavior in terms of stresses is as predicted. [file 13018_2022_2966_MOESM4_ESM.tif]

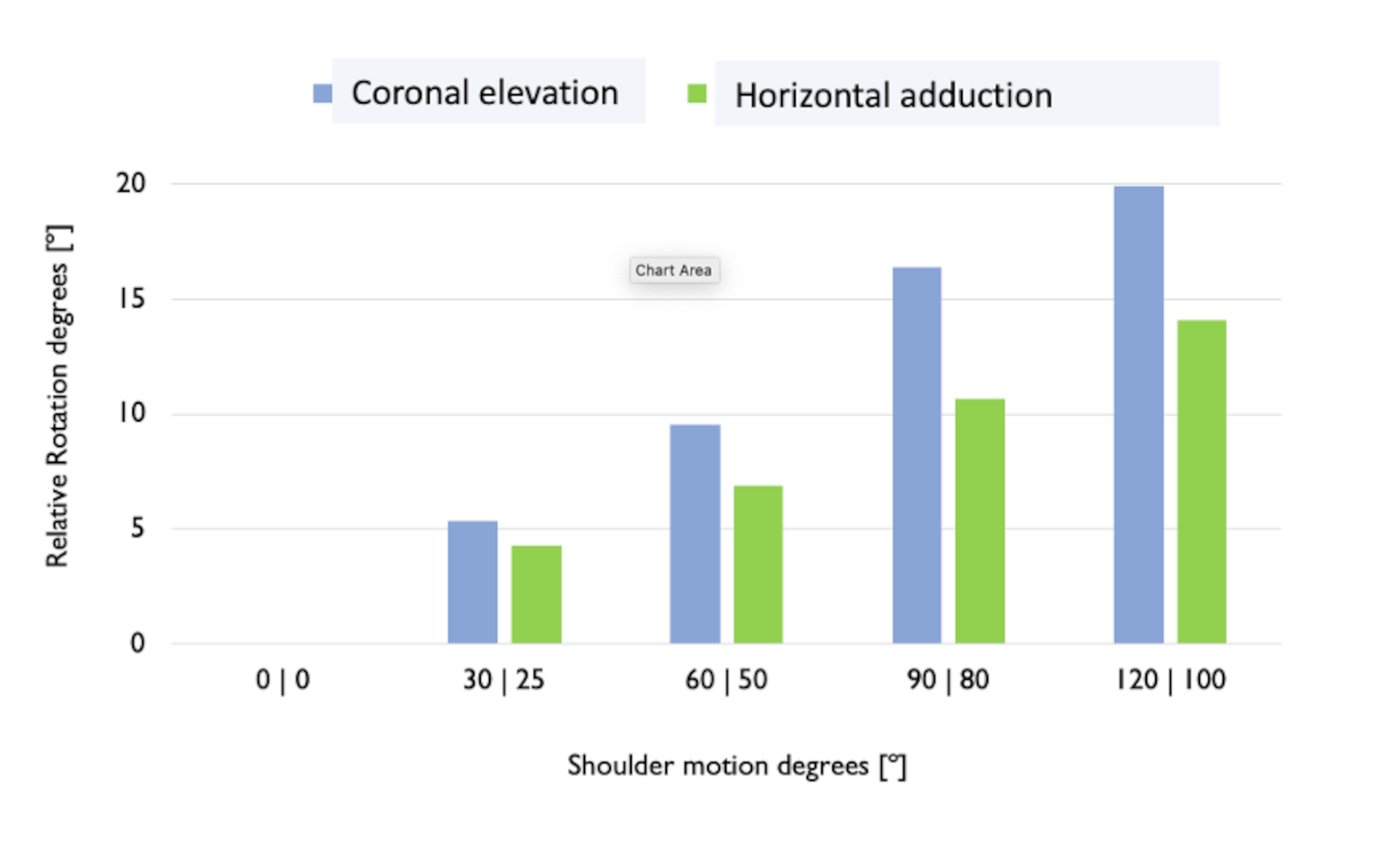

Supplement: Supplementary file 5 — Additional file 5: Acromioclavicular joint relative rotation during shoulder motion.tif. The acromioclavicular joint relative rotation reached 20° at 120° of shoulder elevation in the coronal plane. [file 13018_2022_2966_MOESM5_ESM.tif]
