# Supplementary material for: Anteroinferior bundle of the acromioclavicular ligament plays a substantial role in the joint function during shoulder elevation and horizontal adduction: a finite element model
Source: J Orthop Surg Res. 2022 Feb 5;17:73. doi: 10.1186/s13018-022-02966-0 (PMC8818233; doi:10.1186/s13018-022-02966-0)
Supplement: Supplementary file 3 — Additional file 3: Model validation.pdf. The model was indirectly validated by comparing the kinematic behavior with previously published cadaver biomechanical studies. [file 13018_2022_2966_MOESM3_ESM.pdf]

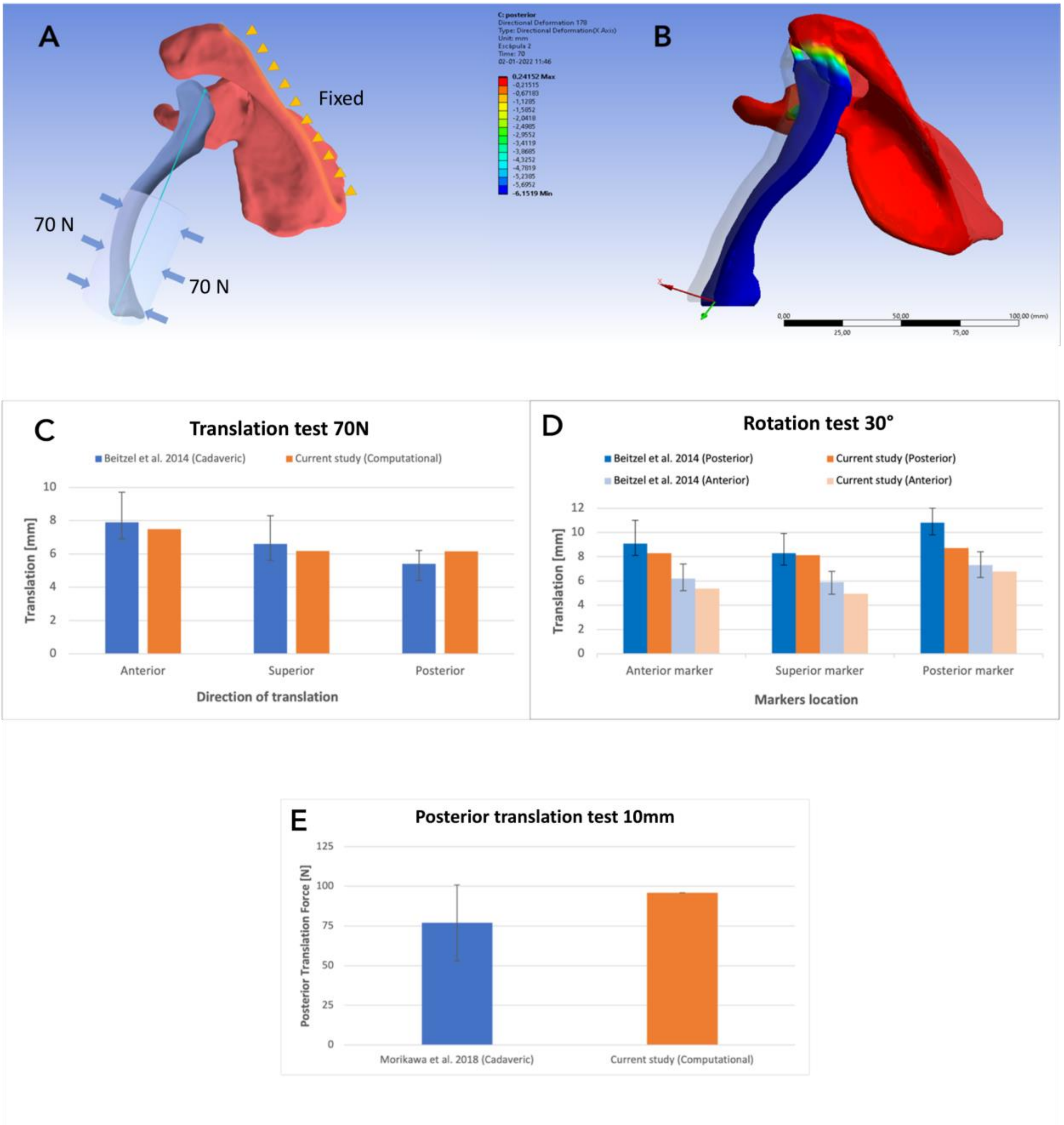

Additional file 3: Model validation. The model was indirectly validated by comparing the kinematic behavior with previously published cadaver biomechanical studies.

a Boundary and loading conditions. Simulation of the setup used by Beitzel et al.[30].
b Posterior translation test. The ghost image shows the original position of the clavicle before applying 70 N of posterior load. The solid image shows the final position of the clavicle.

c Translation test. Under the same loading conditions, the data closely resembled the previously reported normal physiological ranges [30]. The highest percentage difference (12.99 %) was found in the posterior translation (0.75mm).

d Rotation test. The distance of the markers was highly consistent with the translation shown in the cadaver experiments [30]. The highest percentage difference (17.71 %) was found in the superior markers (0.76 mm).

e Posterior translation force. Ninety-six Newtons of posterior force were required to translate the clavicle 10 mm posteriorly in our model, 21.96 % higher than the force measured by Morikawa et al. [10].
